# Why COVID vaccines for young children (5–11 years) are not essential at this moment in time?

**DOI:** 10.1186/s40545-022-00424-0

**Published:** 2022-03-28

**Authors:** Hamid A. Merchant

**Affiliations:** Department of Pharmacy, School of Applied Sciences, University of Huddersfield, HD1 3DH, West Yorkshire, UK

**Keywords:** COVID vaccine, mRNA vaccine, Pfizer BioNTech, BNT162b2, Children, Paediatrics, JCVI, nasal vaccine

## Abstract

The Joint Committee on Vaccination and Immunisation (JCVI) in UK has recently advised that COVID vaccines in 5–11-year-old children is non-essential. This has created an outrage among some healthcare professionals who believed a mandatory vaccination program for all ages would be more beneficial. The JCVI decision sounds strange to many public health professionals in light of the existing practices with regards to other children’s vaccines, for instance flu jabs. The child immunisation should help reduce suffering in children, prevent virus spread in communities, reduce school off days, prevent the loss of quality of life in children and the sufferings from a preventable infection. Therefore, why not support essential COVID vaccines for young children like we do for the flu? This article explains the underlying mechanisms of currently deployed COVID vaccines, the cellular, humoral and mucosal immunity. The article explains why we should not rush mass-immunising young children and a delayed immunisation can be beneficial in offering a more suitable vaccine formulation for children, such as the nasal COVID vaccine, that is going to be available soon and will provide the sought-after protection against infection and transmission,  the public health benefit from the mass immunisation program in children.

## COVID immunisation program in children—what’s the need?

The Joint Committee on Vaccination and Immunisation (JCVI) in UK has recently advised that COVID vaccines (10 mcg Comirnaty) should be offered to 5–11-year-old children in UK, but such immunisation was deemed non-essential by the JCVI [1]. This has created an outrage among some healthcare professionals who believed a mandatory vaccination program for all ages would be more beneficial like in United States and the parts of the Europe.

The JCVI decision sounds strange to many public health professionals in light of the existing practices with regards to other children’s vaccines. Influenza vaccine (flu jabs) for young children, for instance, is a good example. The flu jabs for kids helps reduce children suffering, virus spread in communities, school off days, helps to prevent the loss of quality of life in children and the sufferings from a preventable infection.

The data from early pandemic suggested that most healthy kids were at the least risks of contracting the virus, severe COVID-19, hospitalisation, admission to intensive care or death from COVID-19 [16, 17]. However, as the variants emerged, SARS-CoV-2  did infect children [19], it caused suffering, corroborated community spreads, put vulnerable in family and community at risk [20, 21], affected kids learning and education, risked risk school closures [22] etc.—so why not support essential COVID vaccines for young children like we do for the flu?

Currently deployed COVID vaccines were designed using the variant that was prevalent in early 2020 and the virus has significantly mutated since then; the breakthrough cases from recent Omicron outbreak is a good example of the rapidly evolving nature of this virus [23]. The currently deployed novel COVID vaccines may still provide protection against a severe disease, risk of hospitalisation, admission to intensive care and death, albeit not common in children. COVID vaccines will, however, not provide the children the protection from catching the virus in the first instance nor will prevent minor illness; and it will not reduce the risk of community spread either. Furthermore, JCVI noted that over 85% children aged 5–11 years have had contracted COVID-19 in January 2022 during Omicron outbreak in UK and, therefore, have already acquired natural immunity, that will still provide protection against severe disease on future reinfection [1].

## Intramuscular vs. intranasal vaccines—what’s the difference?

The currently deployed COVID-19 vaccines are currently injected intramuscularly and evidenced a strong cellular and humoral immune response to SARS-CoV-2 following injection [24]. COVID-19 is mediated by SARS-CoV-2 which is a respiratory virus and injectable vaccine induced immunity cannot protect immunised subjects from catching the virus in the first instance. Vaccine induced immunity will, however, protect the subject from severe disease and hospitalisation [25] even if the virus manages to survive the natural defences and enters the circulation. The injectable COVID vaccines do not produce mucosal antibodies e.g., IgA [24] that provide the protection at the point of entry for the virus. This is normally the case with all respiratory pathogens and corresponding injectable vaccines, for instance flu. The efficacy of flu vaccine had always been subjected to controversary as the real-world efficacy ranged as low as 30% to 60% in reducing the risk of catching flu [26]. The flu vaccinated subjects usually do catch flu but mostly recover from a minor illness and flu jabs still prevent a severe disease and flu-associated hospitalisation. Injectable flu vaccines, therefore, were not very successful historically in offering the level of protection sought from mass children immunisation campaign. However, since the introduction of nasal flu jabs for school going children (2–17 years) in UK about a decade ago [7, 8], Public Health England were able to achieve the desired outcome from mass flu immunisation in UK, such as preventing children from catching the infection in the first instance, blocking community transmission that has also reduced the adverse impact on children education and schools etc., boosting the vaccine efficacy to over 87% in 2019 [27].

Several clinical trials confirmed the superior efficacy of nasal flu vaccine over the injectable flu vaccine in preventing infections in children from as young as 6 months to 17 years [18]. In influenza season 2002–2003, studies D153–P514 and D153–P515 in Europe [28] involving 2,085 (6 months to 6 years) and 2,211 children (6–17 years) resulted in 52.7 and 34.7% fewer flu cases than the injectable group, respectively. Later, during 2004–2005 influenza season, the study MI-CP111 [29] involving 7,852 children (aged 6 months to 5 years) across USA, Europe, Asia/Oceania demonstrated 44.5% fewer flu cases than the injectable flu vaccine. Notably, these studies were performed using older generation nasal flu vaccines and more recently a quadrivalent nasal flu  vaccine (Fluenz Tetra) had been rolled out in England that is likely to improve the nasal flu vaccine efficacy even further in comparison to the injectable counterpart.

## Vaccine safety

The other key factor that unbalances the risk/benefit equation of COVID vaccine for the young children is the vaccine safety. The deltoid muscle mass in young children varies significantly across age group and ethnicity and it is not easy to standardise the dose and administration technique among such a heterogenous population. For this reason, very young children with minimal deltoid mass are often contraindicated for intramuscular injections including some vaccines in the arm and instead offered injections in the gluteal muscles. The size of muscle mass often better correlates with the body weight or the BMI than the age. The injectable paediatric vaccine dosages, therefore, cannot be simply based on age as children from 5 to 11 years is a very heterogenous group, and one size would not fit well to everyone when it comes to vaccine safety. The novel COVID vaccines (mRNA or viral vector) are very different to the conventional vaccines (e.g., flu jab). The currently deployed COVID vaccines (mRNA or viral vector) work on the premise of gene delivery and spurs an immune response against vaccine transfected cells in our body. The scientific data on vaccine development and post vaccine rollout surveillance (pharmacovigilance) suggests that injected vaccine was able to distribute away from the injection site, potentially transfecting COVID genes in distance tissues and attracting an immune response. The post-vaccine myocarditis in children and adolescent had been a concern in adolescent and children [3, 4]. There have been suggestions that vaccine distribution to the body tissues away from the injection site may be attributed to poor injection technique. Inadvertent injection of COVID-19 vaccine into deltoid muscle vasculature may result in vaccine distribution to distance tissues and consequent adverse reactions [2, 5, 6]. A very high inter-subject variability in deltoid muscle mass in children and heterogeneity in injection techniques among vaccinators potentially increases the safety risk.

## COVID vaccines: are there any intranasal products?

There remains the question for COVID jabs for young children that could provide a significant benefit, i.e., a protection from catching the infection in children in the first instance and preventing the spread in the community. We understand that the currently deployed injectable COVID vaccines are not able to provide this benefit, and therefore, the JCVI decision to declare COVID vaccinations as ‘non-essential’ in this age group is reasonable. However, there are number of nasal COVID vaccine candidates that are likely to provide the desired benefits and can, therefore, drastically improve the risk/benefit ratio to support mass immunisation campaigns in young children.

Several intranasal COVID vaccine products progressed to clinical evaluation a while ago. Notably, there are at least two nasal COVID vaccine candidates emerging from UK, one from Lancaster university [9] and the other from AstraZeneca [10]. Worth noting that nasal flu vaccine being used in national flu immunisation program by Public Health England is also an AstraZeneca’s product (Fluenz Tetra) and they have the expertise and previous success in producing nasal vaccines. The other nasal COVID vaccine candidates under development include products from CanSinoBIO [11], Bharat Biotech [12] and Sputnik V [13] (all three had their injectable COVID vaccines successfully deployed across the world). In addition, there is a candidate from National Taiwan University Hospital under developmet [14], and yet another nasal vaccine  is jointly developed by the lnfectiology and Public Health (ISP) research unit and the Université de Tours in France [15].

## Conclusions

The nasal COVID vaccines have already evidenced to provide the mucosal antibodies (IgA), i.e., the immune protection in the upper respiratory tract and are likely to protect the children from contracting the virus in the first instance and, therefore, will also prevent community transmission. Moreover, nasal route of delivery will have much safer profile when it comes to adverse events associated with injectable vaccines as it will minimise the risks of vaccine distribution to distant tissues post administration. The nasal delivery is also cost-effective for mass immunisation campaigns in children, it also offers an improved children and parents’ compliance. Therefore, there is no rush to mass vaccinate young children, who are not at the risk of severe disease, using currently available injectable COVID vaccines. Any rushed decision on Covid vaccine mandates in children may also undermine public confidence on vaccines in general and may have adverse implications in childhood immunisation programs, as we already started to see an adverse impact on MMR immunisation rates in United Kingdom.

## References

[1] JCVI statement on vaccination of children aged 5 to 11 years old. Department of Health and Social Care, Government of UK, published 16th Feb 2022. https://www.gov.uk/government/publications/jcvi-update-on-advice-for-covid-19-vaccination-of-children-aged-5-to-11/jcvi-statement-on-vaccination-of-children-aged-5-to-11-years-old. Accessed 22 March 2022.

[2] Merchant H (2021). Inadvertent injection of COVID-19 vaccine into deltoid muscle vasculature may result in vaccine distribution to distance tissues and consequent adverse reactions. Postgrad Med J.

[3] Merchant H (2021). Myocarditis followed by CoViD-19 vaccines: a cause for concern or a reversible minor effect? BMJ 2021;373:n1244/rr-10, https://www.bmj.com/content/373/bmj.n1244/rr-10

[4] Diaz et al., (2021). Myocarditis and Pericarditis After Vaccination for COVID-19. JAMA. 2021;326(12):1210–1212. https://jamanetwork.com/journals/jama/fullarticle/2782900 10.1001/jama.2021.13443 PMC8340007 34347001

[5] Merchant H (2021). Autoimmune damage to the nerves following Covid vaccines: EMA issued warning to patients and healthcare professionals. BMJ 2021;374:n1786/rr-0. https://www.bmj.com/content/374/bmj.n1786/rr-0 10.1186/s40545-021-00315-w PMC7988638 33761987

[6] Merchant H (2021). Might post-injection distribution of CoViD vaccines to the brain explain the rare fatal events of cerebral venous sinus thrombosis (CVST)? BMJ 2021;373:n958/rr-1 https://www.bmj.com/content/373/bmj.n958/rr-1

[7] Flu vaccine to be given to all UK children. Guardian, 25 July 2012. Available at: https://www.theguardian.com/society/2012/jul/25/flu-vaccine-all-uk-children. Accessed 22 March 2022.

[8] Hawkes N (2016). UK stands by nasal flu vaccine for children as US doctors are told to stop using it. BMJ.

[9] Lancaster University intranasal vaccine offers promise to block COVID-19 where it starts. Lancaster University, 10th August 2021. Last accessed on 22nd March 2022 at https://www.lancaster.ac.uk/news/lancaster-university-intranasal-vaccine-offers-promise-to-block-covid-19-where-it-starts

[10] Rubin, R (2021). COVID-19 Vaccine Nasal Spray. JAMA. 2021;326(12):1138. https://jamanetwork.com/journals/jama/fullarticle/2784523 10.1001/jama.2021.14996 34581751

[11] McGregor G (2021). Chinese pharma company is starting trials of an inhaled COVID-19 vaccine. Fortune, April 19, 2021. Last accessed on 22nd March 2022 at https://fortune.com/2021/04/19/covid-19-vaccine-china-cansino-inhale-nasal-spray/

[12] BBV154 - a novel adenovirus vectored, intranasal vaccine for COVID-19. Bharat Biotech, last accessed on 22nd March 2022 at https://www.bharatbiotech.com/intranasal-vaccine.html

[13] The Covid Vaccine We Need Now May Not Be a Shot. The New York Times, 2nd February 2021. Last accessed: 22nd March 2022 at https://www.nytimes.com/2022/02/02/health/covid-vaccine-nasal.html

[14] Smith N and Styllis G (2021). Taiwan joins race to develop nasal vaccine as trials suggest it could be more effective than a jab. Telegraph, 7th October 2021. Last accessed: 22nd March 2022 at https://www.telegraph.co.uk/global-health/science-and-disease/taiwan-joins-race-develop-nasal-vaccine-trials-suggest-could/.

[15] A 100% French nasal vaccine against COVID-19 yields positive pre­-clinical results. INRAE, France, 17 September 2021. Last accessed: 22nd March 2022 at https://www.inrae.fr/en/news/100-french-nasal-vaccine-against-covid-19-yields-positive-pre-clinical-results.

[16] Chappell et al., (2022). Immunocompromised children and young people are at no increased risk of severe COVID-19. Journal of Infection 84 (2022) 31–39. https://www.sciencedirect.com/science/article/pii/S016344532100548X. 10.1016/j.jinf.2021.11.005 PMC8590622 34785268

[17] Merchant HA, Kow CS, Hasan SS (2021). COVID-19 first anniversary review of cases, hospitalization, and mortality in the UK. Expert Rev Respir Med.

[18] Fluenz Tetra nasal spray suspension Influenza vaccine (live attenuated, nasal). Summary of product characteristics, AstraZeneca. Last accessed: 22nd March 2022 at: https://www.medicines.org.uk/emc/medicine/29112.

[19] Hyde Z (2020). COVID-19, children and schools: overlooked and at risk. Med J Aust.

[20] Bhuiyan MU, Stiboy E, Hassan MZ, Chan M, Islam MS, Haider N, Jaffe A, Homaira N (2021). Epidemiology of COVID-19 infection in young children under five years: a systematic review and meta-analysis. Vaccine.

[21] Williams PCM, Howard-Jones AR, Hsu P, Palasanthiran P, Gray PE, McMullan BJ, Britton PN, Bartlett AW (2020). SARS-CoV-2 in children: spectrum of disease, transmission and immunopathological underpinnings. Pathology.

[22] Keeling MJ, Tildesley MJ, Atkins BD, Penman B, Southall E, Guyver-Fletcher G, Holmes A, McKimm H, Gorsich EE, Hill EM, Dyson L (2021). The impact of school reopening on the spread of COVID-19 in England. Phil Trans R Soc B..

[23] Investigation of SARS-CoV-2 variants of concern: technical briefings. Public Health England, 17th September 2021. Last accessed: 22nd March 2022 at: https://www.gov.uk/government/publications/investigation-of-novel-sars-cov-2-variant-variant-of-concern-20201201.

[24] Munro APS, Janani L, Cornelius V, Aley PK, COV-BOOST study group (2021). Safety and immunogenicity of seven COVID-19 vaccines as a third dose (booster) following two doses of ChAdOx1 nCov-19 or BNT162b2 in the UK (COV-BOOST): a blinded, multicentre, randomised, controlled, phase 2 trial. Lancet.

[25] Preliminary data indicate COVID-19 vaccines remain effective against severe disease and hospitalisation caused by the Omicron variant. European Medicines Agency, 11th January 2022. Last accessed: 22nd March 2022 at: https://www.ema.europa.eu/en/news/preliminary-data-indicate-covid-19-vaccines-remain-effective-against-severe-disease-hospitalisation.

[26] Influenza vaccine effectiveness. The European Centre for Disease Prevention and Control, European Union. Last accessed: 22nd March 2022 at https://www.ecdc.europa.eu/en/seasonal-influenza/prevention-and-control/vaccine-effectiveness.

[27] Children’s vaccine 87% effective against circulating flu strain. Public Health England, 22nd February 2019. Last accessed: 22nd March 2022 at https://www.gov.uk/government/news/childrens-vaccine-87-effective-against-circulating-flu-strain.

[28] Trial to Compare the Safety,Tolerability and Efficacy of Influenza Virus Vaccine, (CAIV-T) With Influenza Virus Vaccine, Trivalent, Inactivated (TIV) in Children. ClinicalTrials.gov Identifier: NCT00192205. https://clinicaltrials.gov/ct2/show/NCT00192205.

[29] Trivalent, Live, Cold Adapted Influenza Vaccine (CAIV-T) Against Inactivated Influenza Vaccine (TIV) in Children 6-59 Months of Age. ClinicalTrials.gov Identifier: NCT00128167. https://www.clinicaltrials.gov/ct2/show/NCT00128167.

